# Use of Proton Pump Inhibitors With Dexamethasone in Patients With COVID-19 Pneumonia: Contributions to Long-Term Polypharmacy

**DOI:** 10.7759/cureus.24661

**Published:** 2022-05-02

**Authors:** Ranbir Singh, Megha Kothari

**Affiliations:** 1 Internal Medicine, NewYork-Presbyterian Brooklyn Methodist Hospital, Brooklyn, USA; 2 Gastroenterology, NewYork-Presbyterian Brooklyn Methodist Hospital, Brooklyn, USA

**Keywords:** covid-19, adult gastroenterology, gastroenterology, polyphramacy, dexamethasone, proton pump inhibitor

## Abstract

Introduction

Proton pump inhibitors have been used in conjunction with dexamethasone to treat patients with COVID-19. This is given prophylactically to anticipate possible complications while on steroids, including abdominal pain, gastric ulcers, or gastrointestinal bleeding. Proton pump inhibitors have complications, including *Clostridium difficile *infection, pneumonia, osteoporosis, and vitamin deficiency. The goal of the following project is to assess if there are any subjective and objective benefits to be treated with this regimen instead of dexamethasone on its own. Another inquiry that will be investigated is if this combination results in more patients being prescribed a proton pump inhibitor on discharge.

Materials and methods

The following is a retrospective study involving two groups, the first group taking the aforementioned regimen and the second group on dexamethasone only. Objective findings that will be compared include the change in hemoglobin, blood urea nitrogen, and creatinine levels from admission to discharge between the two groups. Subjective findings include complaints of abdominal pain and reported bloody bowel movements. Medication reconciliation on discharge will also be assessed to observe if patients were discharged with a proton pump inhibitor and how long were they taking this medication as an outpatient.

Results

The difference between hemoglobin, blood urea nitrogen, and creatinine between the two groups was not significant as the p-values were .14, .43. and .10, respectively. Therefore, the null hypothesis was accepted that there was no difference in these objective findings between the two populations. In addition, neither set had complaints of abdominal pain. For the investigated population on a proton pump inhibitor, it was found that 53% of these patients were discharged with this medication. This subset was on this medication for an average of three months, with the maximum duration being seven months for one patient. The data supported the hypothesis that there was no subjective or objective benefit to being on this drug combination, and consequentially, most patients continue to take this medication for months after discharge.

Conclusion

The data affirms the hypothesis that most patients can tolerate dexamethasone without the need for a proton pump inhibitor. This study was limited to patients without any history of gastritis, peptic ulcer disease, or gastrointestinal bleeding; a separate study would need to be done to investigate the need for prophylaxis for patients with these comorbidities. The concerning finding was that patients are being discharged with a medication that they do not need, some patients are taking proton pump inhibitors for more than half the year. There should be further screening to determine if a patient needs to be on a proton pump inhibitor other than steroids.

## Introduction

Glucocorticoids are essential medications used to treat inflammatory, allergic, immunologic, and malignant diseases. Recent results from the RECOVERY trial revealed that treatment with dexamethasone (Dex) reduced the 28-day mortality rate among COVID-19 patients who required supplemental oxygen [1]. However, glucocorticoids are notorious for their adverse effects, including gastritis, ulcer formation, and gastrointestinal bleeding. Previous studies have estimated the relative risk of developing one or more of these gastrointestinal adverse effects as varying from 1.1 (not significant) to 1.5 (marginally significant) [2,3]. Largely in response to these concerns, proton pump inhibitors (PPIs) are frequently prescribed prophylactically to patients treated with glucocorticoids to prevent ulcer formation. Patients are typically provided with this medication for a maximum of 10 days as recommended by the National Institutes of Health [4]. In this study, we examined a series of patients hospitalized for COVID-19 pneumonia to identify any subjective or objective benefits of prophylactic PPI in patients on glucocorticoid therapy. We also determined whether these patients are prescribed PPI on discharge as this may contribute to undesirable polypharmacy.

## Materials and methods

The project was reviewed and approved by the Institutional Review Board of the New York-Presbyterian (NYP) Brooklyn Methodist Hospital (approval number 1884583-3). This was a retrospective study that included 200 patients who were hospitalized for COVID-19 pneumonia and treated with dexamethasone at our facility. The patients were discovered via the electronic medical record system by chart-checking patient admissions between January 2020 to February 2021. The patients identified by our review were divided into two groups. The first group included patients treated with 6 mg of Dex intravenous and 40 mg of pantoprazole orally daily during their hospital stay, and the second group included patients who were treated with Dex only without a PPI. Patients treated with non-steroidal anti-inflammatory drugs and those with a past medical history of peptic ulcer were excluded, as they were already at an increased risk of developing gastrointestinal complications [2]. The patient charts were reviewed for age, gender, and then further evaluated for objective and subjective signs of gastrointestinal bleeding. The length of hospital stay and values for hemoglobin (Hb), blood urea nitrogen (BUN), and creatinine (Cr) at or near the time of patient discharge were compared to those reported on admission as potential objective signs. New-onset abdominal pain was reviewed and compared between the two groups as potential subjective signs. The average differences in these values were compared between the two groups using a two-sample t-test, with a statistical significance of p < 0.05. Charts were also reviewed to determine if the patient needed to return to the emergency department or was readmitted for gastritis, peptic ulcer, or gastrointestinal bleeding within six months of discharge. Ongoing use of a PPI as an outpatient was also noted. The null hypothesis was that there would be no significant differences in subjective or objective measures of gastrointestinal complications between patients treated for COVID pneumonia with Dex and a PPI and those treated with Dex alone. No patients were contacted as part of this project as a chart review was deemed sufficient. 

## Results

We enrolled 200 COVID-19 patients in our study who were admitted in the months of January and February 2021. As shown in Table 1, 100 patients were treated with Dex and a PPI, while 100 were treated with Dex alone. The average age, lengths of hospital stay, and the proportion of male to female patients were similar in both groups. The length of Dex treatment varied between the two groups with the average duration in the steroid and PPI group being eight days and the patients only treated with steroids being six days. None of the patients from either group experienced new-onset abdominal pain during their admissions. Only one patient (0.5%) from the group that was treated with Dex alone was readmitted to the hospital for concerns of new-onset melena for the past four days prior to readmission. This patient was 67-years-old at the time, not discharged with a PPI or Dex, and was readmitted for melena approximately one month after discharge which was concluded to be a result of recent steroid intake. Thus, of the 100 patients treated with Dex alone, only one (1%) developed a significant complication. These findings suggest that PPIs are not necessary for most COVID-19 patients treated with steroids for at most eight days.

**Table 1:** Patient demographics and the number of abdominal complaints recorded in the chart Dex: Dexamethasone; PPI: Proton pump inhibitor

	Treated with Dex and PPI	Treated with Dex alone
Age (years; mean ± SD)	68 ± 17	66 ± 17
Average Hospital Stay (days; mean ± SD)	11 ± 7	9.5 ± 7
Males (n)	49	44
Females (n)	51	56
Totals (n)	100	100
The average duration of dex treatment (days)	8	6
Number of complaints of new-onset abdominal pain while hospitalized	0	0
Number of patients readmitted for possible gastrointestinal bleeding	0	1

Table 2 documents differences in laboratory values from the time of admission to the time of discharge. p-values were determined using two-sample t-tests; ΔHb, change in hemoglobin level; ΔBUN, changes in blood urea nitrogen level; ΔCr, change in creatinine level. As shown, the average change in Hb levels between the two groups was only 0.1 g/dL; this difference did not achieve statistical significance (p = 0.14). Also shown are the average changes in BUN and Cr levels. As in the case of Hb, no statistically significant differences were observed.

**Table 2:** The difference in laboratory values for all patients (N= 200) between hospital admission and discharge +Dex: Treated with Dexamethasone; PPI: Proton pump inhibitor

	+Dex +PPI	+Dex only	P-value
ΔHb (g/dL; mean ± SD)	0.7 ± 0.5	0.6 ± 0.2	0.14
ΔBUN (mg/dL; mean ± SD)	7.8 ± 3.0	8.7 ± 8.9	0.43
ΔCr (mg/dL; mean ± SD)	0.34 ± 0.3	0.24 ± 0.1	0.10

Very few patients experienced abdominal pain and there were no significant differences with respect to the changes in Hb, BUN, or Cr over time. These findings suggest that patients with COVID-19 pneumonia and no history of gastrointestinal bleeding who are managed with Dex may not require concomitant treatment with a PPI. This drug combination also appears to provide no long-term benefit, as only one patient who was only on Dex required readmission for concerns of melena. 

The findings presented in Table 3 document inpatient vs outpatient PPI administration in patients hospitalized with COVID-19. As shown, 33 of the 100 patients (33%) treated with a PPI remained on this drug inpatient for an average of six days after Dex had been discontinued.

**Table 3:** PPI management while in hospital and after discharge. PPI: Proton pump inhibitor; Dex: Dexamethasone

	Values
Total number of patients (N)	200
Number of patients treated with a PPI (n)	100
Number of patients discharged on a PPI (n)	53
Average duration of PPI use (months)	3
Maximum duration of PPI use (months)	7
Number of patients remaining on PPI after discontinuation of Dex use (n)	33
Average duration of PPI use after discontinuation of Dex (days)	6

In a separate finding, fifty-three percent of all patients treated with a PPI while still on Dex were discharged with pantoprazole 40 mg orally daily as an outpatient and continued on this regimen for an average of three months, to a maximum of seven months. Together with the results shown in Table 2, these findings suggest that a PPI may not be necessary for the safe and effective management of COVID-19 pneumonia with Dex and appears to be causing patients to be discharged with PPIs.

## Discussion

During the COVID-19 pandemic, many physicians treated hospitalized patients with Dex to prevent uncontrolled inflammation and then prescribed a PPI prophylaxis to prevent gastrointestinal complications. Our goal in this study was to determine whether the addition of a PPI to steroid treatment was more effective at preventing gastrointestinal complications. We enrolled 200 patients who were divided into two groups of 100 patients each. The two groups had a similar age range, length of hospital stay, and male to female ratio; the data were matched as closely as possible. None of these patients enrolled in our study presented with a history of gastritis or gastrointestinal bleeding. The changes in hemoglobin, BUN, and creatinine levels from admission to discharge were not significantly different between the two groups and neither patient complained about abdominal pain during their hospitalization. Thus, both subjective and objective criteria suggest that these patients were unlikely to be developing symptoms of gastritis. These findings suggest that the addition of a PPI to the drug regimen provides no significant benefit. This hypothesis is also supported by the observation that only one patient was readmitted in one month for melena which was concluded to be due to Dex. Collectively, these data suggest that inpatient PPI prophylaxis provides no long-term benefits as opposed to treatment with Dex alone. 

Previous studies have suggested that PPI is overprescribed both in the hospital and after discharge. In this study, 33% of the patients provided with this drug remained on PPI therapy during their entire hospital stay and remained on board for an average of six days after Dex had been discontinued. Likewise, 53% of the patients remained on PPI therapy for an average of three months after discharge. As we previously discussed, we note that there were no significant short or long-term improvements, either subjectively or objectively, among patients prescribed PPIs prophylactically to prevent the negative sequelae of steroids. PPIs are typically prescribed for two months at most for patients with gastritis and peptic ulcer disease with endoscopic intervention. Within this timeframe, PPIs are discontinued if there is no symptomatic improvement as described by the American College of Gastroenterology (ACG) [5]. These findings suggest that the ongoing use of PPI in this manner supports the hypothesis that this method of prescribing PPI with Dex is contributing to overprescription of PPI and polypharmacy. 

PPI overprescribing has emerged as a critical issue in the past decade. This topic was highlighted in a report by Metaxas et al. who discussed PPI overuse among US veterans [6]. Automation in medicine is likely to be a major source of this problem nationwide. Most electronic medical record systems will automatically renew both inpatient and outpatient prescriptions without substantial input from the prescribing physician. While this saves time for medical professionals, a major drawback is that medications that are no longer needed will be continued more or less unchecked. There is not much research on the drawbacks associated with medical automation. This relatively new phenomenon should be the subject of further investigation. 

Physicians may feel pressured by patients and other healthcare providers to prescribe PPIs prophylactically and may be instructed to do so by their superiors. Several physicians on the same team may prescribe related PPIs; this can clearly result in polypharmacy due to multiple physicians and consultants managing the same patient. As shown in the study, PPIs provide no short or long-term benefits for steroid-treated patients who have no history of gastritis or peptic ulcer disease. Furthermore, once patients are provided with a prescription for a PPI on discharge, it may be difficult to discontinue it as an outpatient. As noted by Halli-Tierney et al. patients may ask their primary care physicians to continue prescribing multiple medications that they perceive as providing some benefit [7]. This may be due to an overall limited understanding of the specific benefits, and the concern that, without each of these medications, their troubling symptoms might return. Simply providing prescriptions for these medications may help streamline the outpatient visit and the clinic workflow, as health care providers may not have all the additional time needed to discuss the specific drawbacks of multiple medication regimens.

Several studies have highlighted the process in which patients are treated with PPIs as an inpatient and are then provided with prescriptions that are sent to their pharmacy on discharge. For example, Pham et al. reported that 54% of the 213 patients enrolled in their retrospective study were treated with a PPI as an inpatient for no clear indication and were instructed to continue taking this medication for at least six months as an outpatient [8]. In another retrospective study, Grant et al. [9] found that 60% of the 58 patients were appropriately treated with a PPI and that 86% of the patients in this group were discharged on this medication, with 62% of these patients remaining on this medication for more than six months. However, among the patients treated inappropriately with a PPI, 65% of them were discharged with a prescription and 71% of these patients remained on this medication for more than six months. Collectively, these studies underscore the frequency and extent of inappropriate use of PPI, most notably on hospital discharge.

The COVID-19 pandemic most likely increased the frequency of PPI prescriptions provided to patients on discharge from the hospital. Overprescribing can be harmful to the patient and is costly to the American healthcare system. Heidelbaugh et al. reported that the total cost of inappropriate PPI use in 2010 was somewhere between $230,000 and $1,500,000 based on average wholesale prices [10]. This medication has also been associated with serious adverse effects including *Clostridium difficile*-associated diarrhea, community-acquired pneumonia, microscopic colitis, osteoporosis, and vitamin B12 deficiency [11,12]. While the number of patients experiencing these adverse events may have increased in parallel with the number of PPI prescriptions provided during the pandemic, it will take more time to assess the existence and severity of this association.

The best way to address this problem is to consider the full clinical picture of each patient before prescribing prophylactic mediations. Patients with known risk factors including a past history of gastritis, peptic ulcer, or gastrointestinal bleeding who require Dex treatment would certainly be candidates for PPI prophylaxis [2]. Medication reconciliation on discharge is another important way to prevent polypharmacy. Likewise, while medical automation helps to save time and resources, this may permit patients to remain on unnecessary medication for months to years. Healthcare professionals need to perform an accurate medication reconciliation to help prevent overprescription of PPIs as well as other drugs of concern.

This study has several limitations, most notably a small sample size (200 patients). These results will require confirmation in a larger patient cohort. Furthermore, the demographic data were not completely matched; the two groups had patients of varying ages and lengths of hospital stay which could have an impact on their risk of developing gastrointestinal symptoms. Geography was also a limitation in this case. The study was limited to patients who were hospitalized at NYP-Brooklyn Methodist Hospital which is located in Brooklyn, NY. Other hospitals may have different protocols for managing inpatient PPI use. Similarly, we only had access to patient medical records from this single hospital. We have no way of knowing if any of our patients were admitted to another hospital after discharge from NYP-Brooklyn Methodist for evaluation or treatment of potential gastrointestinal bleeding. 

## Conclusions

Many patients with COVID-19 pneumonia were managed with Dex together with a PPI. The PPI was prescribed prophylactically to prevent potential gastrointestinal complications of steroid use including gastritis, peptic ulcer disease, and gastrointestinal bleeding. The results of the study suggest that there is no clinical benefit to including a PPI in this patient population. While this may seem innocuous, this can lead to patients remaining on PPIs longer than anticipated; some patients will be provided with a prescription for a PPI on discharge and will continue to take this drug for many months. The pandemic seems to have exacerbated and accelerated the pre-existing problem of PPI overprescription. Healthcare professionals must assess the specific risk in each patient before prescribing a PPI and must remember to discontinue this medication when it is no longer needed to prevent ongoing polypharmacy. 
